# Tetra­aqua­bis{μ_2_-2,7-bis­[(2,6-diisopropyl­phen­yl)imino­meth­yl]naphthalene-1,8-diolato}di-μ_3_-hydroxido-di-μ_2_-hydroxido-bis­(trimethyl­phosphine oxide)tetra­nickel(II)–trimethyl­phosphine oxide–diethyl ether–water (1/2/2/2)

**DOI:** 10.1107/S1600536810003697

**Published:** 2010-02-06

**Authors:** Massimiliano Delferro, Michael P. Weberski, Brandon A. Rodriguez, Tobin J. Marks

**Affiliations:** a Department of Chemistry, Northwestern University, Evanston, IL 60208-3113, USA

## Abstract

The title complex, [Ni_4_(C_36_H_40_N_2_O_2_)_2_(OH)_4_(C_3_H_9_OP)_2_(H_2_O)_4_]·2C_4_H_10_O·2C_3_H_9_OP·2H_2_O, is centrosymmetric with a central core that can be described as a defect double cubane. The four metal ions in the cluster are held together by four bridging hydroxide groups. Each Ni^II^ atom adopts a distorted octa­hedral geometry.

## Related literature

For neutral bimetallic nickel catalysts for ethyl­ene polymerizations and co-polymerizations with polar monomers, see: Rodriguez *et al.* (2008 ▶, 2009 ▶). For bond-valence-sum calculation, see: Brese & O’Keeffe (1991 ▶). For related structures, see: King *et al.* (2004 ▶). For Ni⋯Ni inter­actions, see: Soldatov *et al.* (2001 ▶).
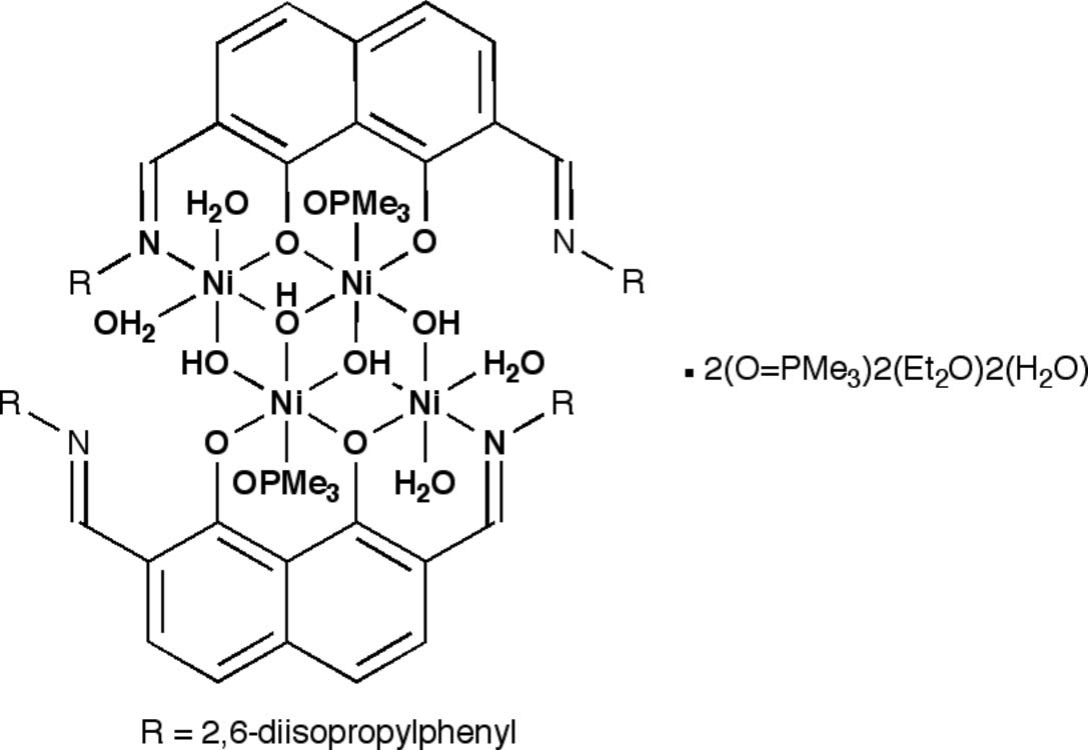


## Experimental

### 

#### Crystal data

[Ni_4_(C_36_H_40_N_2_O_2_)_2_(OH)_4_(C_3_H_9_OP)_2_(H_2_O)_4_]·2C_4_H_10_O·2C_3_H_9_OP·2H_2_O
*M* *_r_* = 1992.90
Triclinic, 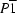

*a* = 11.9818 (2) Å
*b* = 13.7937 (2) Å
*c* = 16.2215 (3) Å
α = 78.109 (1)°
β = 72.759 (1)°
γ = 83.782 (1)°
*V* = 2502.37 (7) Å^3^
*Z* = 1
Cu *K*α radiation
μ = 1.99 mm^−1^
*T* = 100 K
0.28 × 0.16 × 0.10 mm

#### Data collection

Bruker APEXII CCD diffractometer
Absorption correction: multi-scan (*SADABS*; Bruker, 2001 ▶) *T*_min_ = 0.787, *T*_max_ = 0.898
27203 measured reflections
8609 independent reflections
6279 reflections with *I* > 2σ(*I*)
*R*_int_ = 0.050

#### Refinement

*R*[*F* ^2^ > 2σ(*F* ^2^)] = 0.046
*wR*(*F* ^2^) = 0.162
*S* = 1.00
8609 reflections
575 parameters
H-atom parameters constrained
Δρ_max_ = 0.79 e Å^−3^
Δρ_min_ = −0.89 e Å^−3^

### 

Data collection: *APEX2* (Bruker, 2004 ▶); cell refinement: *SAINT* (Bruker, 2001 ▶); data reduction: *SAINT*; program(s) used to solve structure: *SHELXS97* (Sheldrick, 2008 ▶); program(s) used to refine structure: *SHELXL97* (Sheldrick, 2008 ▶); molecular graphics: *ORTEP-3 for Windows* (Farrugia, 1997 ▶); software used to prepare material for publication: *WinGX* (Farrugia, 1999 ▶).

## Supplementary Material

Crystal structure: contains datablocks I, global. DOI: 10.1107/S1600536810003697/rz2413sup1.cif

Structure factors: contains datablocks I. DOI: 10.1107/S1600536810003697/rz2413Isup2.hkl

Additional supplementary materials:  crystallographic information; 3D view; checkCIF report

## Figures and Tables

**Fig. 1.**
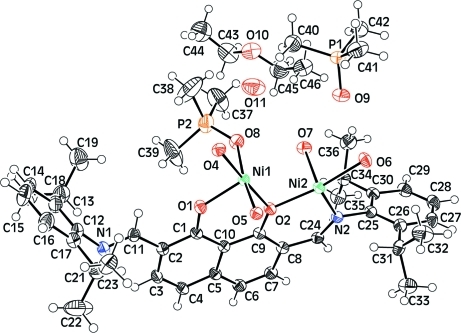
The asymmetric unit of the title compound, with displacement ellipsoids drawn at the 50% probability level.

**Fig. 2.**
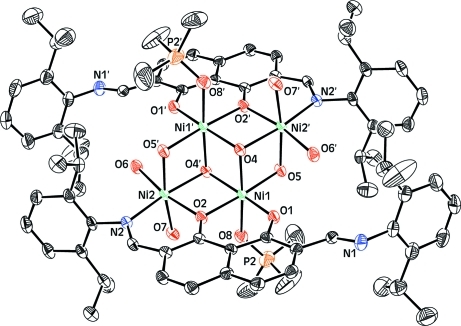
The structure of the title complex molecule. Hydrogen atoms are omitted for clarity. Symmetry code: (') -x, 1-y, 1-z.

## supplementary crystallographic information

### Comment

The X-ray structural determination of the title compound [FI^2^—Ni_2_-(O=PMe_3_)(H_2_O)_2_(µ_2_-OH)(µ_3_-OH)]_2_.2(O=PMe_3_).2(Et_2_O) .2(H_2_O), (*I*), FI^2^ = 2,7-di(2,6-diisopropylphenyl)imino-1,8-napthalenediolato] reveals a tetranuclear nickel complex (Figure 1). This hydrated complex is centrosymmetric with a central core that can be described as a defect double cubane with two missing vertices. The four nickel(II) ions in the cluster are held together by four bridging hydroxide groups. In the defect dicubane core, two types of distorted octahedrally coordinated nickel(II) ions can be observed. Atom Ni1 is coordinated by two µ_3_-OH (O4 and O4'), one µ_2_-OH (O5'), and the FI^2^ ligand in a bidentate chelating mode through the two phenolate oxygen atoms (O1, O2). The nickel coordination is completed by one trimethylphosphine oxide ligand (O8). Atom Ni2 is coordinated by one µ_3_-OH (O4'), one µ_2_-OH (O5), and the FI^2^ ligand in a bidentate chelating mode through the imine nitrogen atom and a phenolate oxygen atom (N2 and O2, respectively). Two water molecules complete the coordination geometry (O6 and O7). The presence of hydroxyl groups and water molecules are confirmed by Bond Valence Sum calculations (0.28 v.u. for H_2_O, 0.68 v.u. for µ_2_-OH and 1.02 v.u. for µ_3_-OH; Brese & O'Keeffe, 1991). Defect dicubane-like structures are common in literature [nickel(II), copper(II), cobalt(II), iron(II) and manganese(II); King *et al.*, 2004 and references herein] and exhibit similar bond distances and angles with respect to the present product. In I the intramolecular Ni..Ni distances range from 3.052 (1) to 3.073 (4) Å. These values are shorter than the sum of Ni atomic van der Waals radii (3.3 Å) and may indicate chemically significant interactions (Soldatov *et al.*, 2001 and references herein).

### Experimental

In attempts to grow crystals of {2,7-di-[(2,6-diisopropylphenyl)imino]-1,8-naphthalenediolato}(methyl) (trimethylphosphine)nickel(II) (Rodriguez *et al.*, 2008, 2009) by slow diffusion of hexane into ethyl ether solutions under inert atmosphere, small quantities of yellow block-like crystals of the hydrated dimeric decomposition title product were isolated.

### Refinement

The hydrogen atoms were introduced into the geometrically calculated positions and refined using a riding model with distance C—H = 0.95 Å, *U*_iso_ = 1.2 *U*_eq_ (C) for aromatic atoms and imine groups, C—H = 0.98 Å, *U*_iso_ = 1.5 *U*_eq_(C) for methyl groups, C—H = 0.99 Å, *U*_iso_ = 1.2 *U*_eq_(C) for methylene groups and C—H =1.00 Å, *U*_iso_ = 1.2 *U*_eq_(C) for methine groups. The hydrogen atoms on the hydroxyl groups (O4 and O5) and water molecules (O6, O7 and O11) could not be located in the difference Fourier map or placed geometrically, and therefore were omitted from the refinement. A bond valence sum (BVS; Brese & O'Keeffe, 1991) calculation was applied to determine the valences of these oxygen atoms. The thermal ellipsoids of C20, C37, C38, C39, C40, C41 and C42 atoms are relatively large. We are confident in their assignment based on the chemistry of the molecule.

### Crystal data

**Table:**

[Ni_4_(C_36_H_40_N_2_O_2_)_2_(OH)_4_(C_3_H_9_OP)_2_(H_2_O)_4_]·2C_4_H_10_O·2C_3_H_9_OP·2H_2_O	*Z* = 1
*M**_r_* = 1992.90	*F*(000) = 1052
Triclinic, *P*1	*D*_x_ = 1.314 Mg m^−^^3^
Hall symbol: -P 1	Cu *K*α radiation, λ = 1.54178 Å
*a* = 11.9818 (2) Å	Cell parameters from 9944 reflections
*b* = 13.7937 (2) Å	θ = 5.7–66.9°
*c* = 16.2215 (3) Å	µ = 1.99 mm^−^^1^
α = 78.109 (1)°	*T* = 100 K
β = 72.759 (1)°	Block, yellow
γ = 83.782 (1)°	0.28 × 0.16 × 0.10 mm
*V* = 2502.37 (7) Å^3^	

### Data collection

**Table:**

Bruker APEXII CCD diffractometer	8609 independent reflections
Radiation source: fine-focus sealed tube	6279 reflections with *I* > 2σ(*I*)
graphite	*R*_int_ = 0.050
φ and ω scans	θ_max_ = 67.3°, θ_min_ = 2.9°
Absorption correction: multi-scan (*SADABS*; Bruker, 2001)	*h* = −14→14
*T*_min_ = 0.787, *T*_max_ = 0.898	*k* = −5→16
27203 measured reflections	*l* = −18→19

### Refinement

**Table:**

Refinement on *F*^2^	Primary atom site location: structure-invariant direct methods
Least-squares matrix: full	Secondary atom site location: difference Fourier map
*R*[*F*^2^ > 2σ(*F*^2^)] = 0.046	Hydrogen site location: inferred from neighbouring sites
*wR*(*F*^2^) = 0.162	H-atom parameters constrained
*S* = 1.00	*w* = 1/[σ^2^(*F*_o_^2^) + (0.1064*P*)^2^] where *P* = (*F*_o_^2^ + 2*F*_c_^2^)/3
8609 reflections	(Δ/σ)_max_ = 0.001
575 parameters	Δρ_max_ = 0.79 e Å^−^^3^
0 restraints	Δρ_min_ = −0.89 e Å^−^^3^

### Special details

**Table:**

Geometry. All esds (except the esd in the dihedral angle between two l.s. planes) are estimated using the full covariance matrix. The cell esds are taken into account individually in the estimation of esds in distances, angles and torsion angles; correlations between esds in cell parameters are only used when they are defined by crystal symmetry. An approximate (isotropic) treatment of cell esds is used for estimating esds involving l.s. planes.
Refinement. Refinement of *F*^2^ against ALL reflections. The weighted *R*-factor wR and goodness of fit *S* are based on *F*^2^, conventional *R*-factors *R* are based on *F*, with *F* set to zero for negative *F*^2^. The threshold expression of *F*^2^ > σ(*F*^2^) is used only for calculating *R*-factors(gt) etc. and is not relevant to the choice of reflections for refinement. *R*-factors based on *F*^2^ are statistically about twice as large as those based on *F*, and *R*- factors based on ALL data will be even larger.

### Fractional atomic coordinates and isotropic or equivalent isotropic displacement parameters (Å^2^)

**Table:**

	*x*	*y*	*z*	*U*_iso_*/*U*_eq_	
Ni1	−0.01942 (5)	0.61224 (4)	0.49749 (3)	0.02009 (16)	
Ni2	−0.06318 (5)	0.47043 (4)	0.67392 (3)	0.02006 (16)	
P1	0.52123 (8)	0.45032 (7)	0.68083 (7)	0.0338 (2)	
P2	−0.21683 (10)	0.80223 (7)	0.53249 (7)	0.0454 (3)	
O1	0.0870 (2)	0.72178 (16)	0.44528 (14)	0.0247 (5)	
O2	0.0298 (2)	0.59307 (16)	0.60874 (14)	0.0226 (5)	
O4	0.10543 (19)	0.51166 (15)	0.44142 (13)	0.0216 (5)	
O5	0.0676 (2)	0.37798 (16)	0.61391 (14)	0.0242 (5)	
O6	−0.1683 (2)	0.34634 (17)	0.71743 (15)	0.0280 (5)	
O7	−0.2103 (2)	0.56828 (17)	0.71747 (14)	0.0270 (5)	
O8	−0.1672 (2)	0.70163 (17)	0.55997 (15)	0.0313 (6)	
O9	0.6242 (2)	0.4500 (2)	0.7146 (2)	0.0453 (7)	
O10	0.4306 (3)	0.8467 (2)	0.7711 (2)	0.0512 (8)	
O11	0.2730 (3)	0.4515 (2)	0.5759 (2)	0.0596 (9)	
N1	0.2850 (3)	0.9281 (2)	0.27509 (19)	0.0308 (7)	
N2	−0.0098 (2)	0.47072 (19)	0.78290 (17)	0.0216 (6)	
C1	0.1591 (3)	0.7559 (2)	0.4778 (2)	0.0209 (7)	
C2	0.2307 (3)	0.8329 (2)	0.4222 (2)	0.0233 (7)	
C3	0.3124 (3)	0.8718 (2)	0.4510 (2)	0.0254 (7)	
H3	0.3620	0.9209	0.4117	0.030*	
C4	0.3228 (3)	0.8410 (2)	0.5340 (2)	0.0262 (7)	
H4	0.3779	0.8697	0.5523	0.031*	
C5	0.2518 (3)	0.7663 (2)	0.5931 (2)	0.0235 (7)	
C6	0.2616 (3)	0.7386 (2)	0.6799 (2)	0.0265 (7)	
H6	0.3135	0.7716	0.6979	0.032*	
C7	0.1974 (3)	0.6654 (2)	0.7375 (2)	0.0260 (7)	
H7	0.2050	0.6487	0.7954	0.031*	
C8	0.1192 (3)	0.6129 (2)	0.7143 (2)	0.0228 (7)	
C9	0.1029 (3)	0.6403 (2)	0.6289 (2)	0.0202 (7)	
C10	0.1697 (3)	0.7203 (2)	0.5664 (2)	0.0205 (7)	
C11	0.2181 (3)	0.8691 (2)	0.3343 (2)	0.0245 (7)	
H11	0.1547	0.8466	0.3206	0.029*	
C12	0.2579 (3)	0.9552 (3)	0.1927 (2)	0.0303 (8)	
C13	0.3096 (3)	0.8997 (3)	0.1264 (2)	0.0322 (8)	
C14	0.2836 (4)	0.9286 (3)	0.0472 (3)	0.0429 (10)	
H14	0.3176	0.8916	0.0017	0.051*	
C15	0.2093 (4)	1.0101 (3)	0.0324 (3)	0.0484 (11)	
H15	0.1927	1.0286	−0.0226	0.058*	
C16	0.1596 (4)	1.0640 (3)	0.0974 (3)	0.0448 (10)	
H16	0.1087	1.1200	0.0868	0.054*	
C17	0.1821 (4)	1.0386 (3)	0.1790 (3)	0.0377 (9)	
C18	0.3936 (4)	0.8116 (3)	0.1404 (3)	0.0377 (9)	
H18	0.3729	0.7840	0.2048	0.045*	
C19	0.5170 (5)	0.8457 (4)	0.1131 (6)	0.095 (2)	
H19A	0.5369	0.8796	0.0516	0.143*	
H19B	0.5715	0.7882	0.1195	0.143*	
H19C	0.5228	0.8915	0.1503	0.143*	
C20	0.3867 (4)	0.7282 (3)	0.0936 (3)	0.0449 (10)	
H20A	0.4168	0.7502	0.0300	0.067*	
H20B	0.3050	0.7108	0.1081	0.067*	
H20C	0.4337	0.6700	0.1127	0.067*	
C21	0.1226 (4)	1.0953 (3)	0.2525 (3)	0.0450 (10)	
H21	0.1614	1.0729	0.3004	0.054*	
C22	−0.0050 (5)	1.0727 (4)	0.2899 (4)	0.0651 (14)	
H22A	−0.0454	1.0951	0.2443	0.098*	
H22B	−0.0406	1.1073	0.3392	0.098*	
H22C	−0.0120	1.0011	0.3104	0.098*	
C23	0.1342 (5)	1.2072 (3)	0.2228 (4)	0.0638 (14)	
H23A	0.2171	1.2215	0.1981	0.096*	
H23B	0.0999	1.2402	0.2733	0.096*	
H23C	0.0929	1.2316	0.1781	0.096*	
C24	0.0601 (3)	0.5359 (2)	0.7833 (2)	0.0217 (7)	
H24	0.0750	0.5324	0.8382	0.026*	
C25	−0.0589 (3)	0.4076 (2)	0.8670 (2)	0.0234 (7)	
C26	−0.1445 (3)	0.4479 (3)	0.9331 (2)	0.0255 (7)	
C27	−0.1880 (3)	0.3843 (3)	1.0131 (2)	0.0317 (8)	
H27	−0.2451	0.4098	1.0592	0.038*	
C28	−0.1509 (3)	0.2859 (3)	1.0272 (2)	0.0349 (9)	
H28	−0.1826	0.2443	1.0821	0.042*	
C29	−0.0683 (4)	0.2484 (3)	0.9619 (2)	0.0326 (8)	
H29	−0.0429	0.1805	0.9722	0.039*	
C30	−0.0204 (3)	0.3071 (2)	0.8809 (2)	0.0270 (8)	
C31	−0.1904 (3)	0.5547 (3)	0.9231 (2)	0.0282 (8)	
H31	−0.1555	0.5874	0.8608	0.034*	
C32	−0.1531 (4)	0.6098 (3)	0.9820 (2)	0.0393 (9)	
H32A	−0.1819	0.5766	1.0433	0.059*	
H32B	−0.1858	0.6782	0.9754	0.059*	
H32C	−0.0675	0.6099	0.9653	0.059*	
C33	−0.3232 (4)	0.5627 (3)	0.9411 (3)	0.0436 (10)	
H33A	−0.3456	0.5267	0.9028	0.065*	
H33B	−0.3496	0.6326	0.9295	0.065*	
H33C	−0.3597	0.5339	1.0026	0.065*	
C34	0.0743 (3)	0.2627 (3)	0.8124 (2)	0.0303 (8)	
H34	0.0928	0.3144	0.7578	0.036*	
C35	0.1864 (4)	0.2367 (3)	0.8401 (3)	0.0431 (10)	
H35A	0.2105	0.2954	0.8544	0.065*	
H35B	0.2482	0.2145	0.7919	0.065*	
H35C	0.1724	0.1835	0.8918	0.065*	
C36	0.0362 (4)	0.1729 (3)	0.7892 (3)	0.0506 (11)	
H36A	0.0168	0.1207	0.8416	0.076*	
H36B	0.0999	0.1483	0.7435	0.076*	
H36C	−0.0328	0.1916	0.7677	0.076*	
C37	−0.1105 (5)	0.8943 (3)	0.5018 (5)	0.102 (3)	
H37A	−0.0723	0.8887	0.5484	0.152*	
H37B	−0.1492	0.9604	0.4931	0.152*	
H37C	−0.0517	0.8843	0.4472	0.152*	
C38	−0.2867 (7)	0.8036 (6)	0.4477 (4)	0.103 (3)	
H38A	−0.2271	0.7942	0.3933	0.155*	
H38B	−0.3298	0.8674	0.4378	0.155*	
H38C	−0.3410	0.7499	0.4661	0.155*	
C39	−0.3351 (5)	0.8392 (4)	0.6211 (4)	0.0737 (17)	
H39A	−0.3954	0.7902	0.6407	0.111*	
H39B	−0.3691	0.9043	0.6005	0.111*	
H39C	−0.3049	0.8432	0.6702	0.111*	
C40	0.5061 (4)	0.5604 (3)	0.6042 (3)	0.0459 (10)	
H40A	0.5016	0.6185	0.6313	0.069*	
H40B	0.4345	0.5591	0.5870	0.069*	
H40C	0.5739	0.5641	0.5521	0.069*	
C41	0.5279 (4)	0.3478 (3)	0.6277 (3)	0.0512 (11)	
H41A	0.5975	0.3510	0.5770	0.077*	
H41B	0.4577	0.3504	0.6078	0.077*	
H41C	0.5320	0.2857	0.6690	0.077*	
C42	0.3880 (4)	0.4423 (3)	0.7677 (3)	0.0473 (10)	
H42A	0.3915	0.3815	0.8108	0.071*	
H42B	0.3222	0.4408	0.7437	0.071*	
H42C	0.3771	0.5000	0.7963	0.071*	
C43	0.4650 (5)	0.7806 (4)	0.8388 (3)	0.0617 (13)	
H43A	0.4074	0.7855	0.8961	0.074*	
H43B	0.5421	0.7978	0.8410	0.074*	
C44	0.4726 (5)	0.6766 (4)	0.8215 (4)	0.0640 (14)	
H44A	0.3993	0.6631	0.8118	0.096*	
H44B	0.4858	0.6293	0.8722	0.096*	
H44C	0.5377	0.6697	0.7693	0.096*	
C45	0.4276 (5)	0.9458 (4)	0.7783 (4)	0.0635 (14)	
H45A	0.5075	0.9648	0.7719	0.076*	
H45B	0.3776	0.9553	0.8370	0.076*	
C46	0.3819 (5)	1.0083 (4)	0.7111 (4)	0.0713 (15)	
H46A	0.4268	0.9940	0.6534	0.107*	
H46B	0.3883	1.0782	0.7125	0.107*	
H46C	0.2995	0.9952	0.7220	0.107*	

### Atomic displacement parameters (Å^2^)

**Table:**

	*U*^11^	*U*^22^	*U*^33^	*U*^12^	*U*^13^	*U*^23^
Ni1	0.0213 (3)	0.0235 (3)	0.0156 (3)	−0.0036 (2)	−0.0049 (2)	−0.0030 (2)
Ni2	0.0201 (3)	0.0255 (3)	0.0150 (3)	−0.0046 (2)	−0.0047 (2)	−0.0031 (2)
P1	0.0237 (5)	0.0374 (5)	0.0404 (6)	−0.0022 (4)	−0.0104 (4)	−0.0050 (4)
P2	0.0444 (7)	0.0345 (5)	0.0432 (6)	0.0111 (5)	0.0020 (5)	−0.0041 (4)
O1	0.0294 (13)	0.0263 (12)	0.0199 (11)	−0.0081 (10)	−0.0083 (10)	−0.0017 (9)
O2	0.0241 (13)	0.0269 (11)	0.0182 (11)	−0.0071 (9)	−0.0066 (9)	−0.0035 (9)
O4	0.0219 (12)	0.0266 (11)	0.0174 (11)	−0.0027 (9)	−0.0065 (9)	−0.0041 (9)
O5	0.0245 (13)	0.0316 (12)	0.0170 (11)	0.0007 (10)	−0.0071 (10)	−0.0046 (9)
O6	0.0297 (14)	0.0320 (12)	0.0206 (12)	−0.0115 (10)	−0.0037 (10)	−0.0008 (9)
O7	0.0239 (13)	0.0362 (13)	0.0211 (12)	0.0003 (10)	−0.0045 (10)	−0.0093 (10)
O8	0.0314 (14)	0.0342 (13)	0.0254 (13)	0.0020 (11)	−0.0040 (11)	−0.0065 (10)
O9	0.0325 (16)	0.0483 (16)	0.0595 (19)	−0.0083 (12)	−0.0217 (14)	−0.0038 (13)
O10	0.0483 (19)	0.0586 (19)	0.0521 (19)	0.0042 (14)	−0.0173 (15)	−0.0211 (15)
O11	0.0351 (18)	0.065 (2)	0.079 (2)	0.0031 (15)	−0.0123 (16)	−0.0217 (17)
N1	0.0299 (17)	0.0317 (15)	0.0274 (16)	−0.0074 (13)	−0.0054 (13)	0.0013 (12)
N2	0.0216 (15)	0.0246 (14)	0.0187 (13)	−0.0021 (11)	−0.0060 (11)	−0.0034 (11)
C1	0.0192 (17)	0.0215 (15)	0.0212 (16)	−0.0010 (13)	−0.0024 (13)	−0.0073 (12)
C2	0.0239 (18)	0.0212 (16)	0.0229 (17)	−0.0007 (13)	−0.0034 (14)	−0.0051 (13)
C3	0.0237 (19)	0.0217 (16)	0.0260 (18)	−0.0039 (13)	−0.0006 (14)	−0.0021 (13)
C4	0.0239 (19)	0.0257 (17)	0.0303 (19)	−0.0071 (14)	−0.0067 (15)	−0.0066 (14)
C5	0.0233 (18)	0.0226 (16)	0.0244 (17)	−0.0027 (13)	−0.0046 (14)	−0.0060 (13)
C6	0.0268 (19)	0.0296 (18)	0.0271 (18)	−0.0050 (14)	−0.0106 (15)	−0.0074 (14)
C7	0.0273 (19)	0.0310 (18)	0.0222 (17)	−0.0034 (14)	−0.0102 (15)	−0.0048 (14)
C8	0.0223 (18)	0.0255 (16)	0.0214 (17)	−0.0018 (13)	−0.0053 (14)	−0.0070 (13)
C9	0.0200 (17)	0.0223 (16)	0.0192 (16)	−0.0018 (13)	−0.0045 (13)	−0.0069 (12)
C10	0.0208 (17)	0.0208 (15)	0.0198 (16)	−0.0002 (13)	−0.0041 (13)	−0.0064 (12)
C11	0.0271 (19)	0.0209 (16)	0.0236 (17)	−0.0001 (14)	−0.0051 (15)	−0.0035 (13)
C12	0.032 (2)	0.0290 (18)	0.0259 (18)	−0.0061 (15)	−0.0063 (16)	0.0043 (14)
C13	0.031 (2)	0.0314 (19)	0.031 (2)	−0.0058 (15)	−0.0076 (16)	0.0001 (15)
C14	0.050 (3)	0.045 (2)	0.027 (2)	0.0015 (19)	−0.0072 (19)	−0.0010 (17)
C15	0.066 (3)	0.046 (2)	0.028 (2)	0.000 (2)	−0.017 (2)	0.0069 (17)
C16	0.048 (3)	0.036 (2)	0.041 (2)	0.0060 (18)	−0.012 (2)	0.0090 (18)
C17	0.038 (2)	0.0324 (19)	0.035 (2)	−0.0009 (16)	−0.0042 (18)	0.0027 (16)
C18	0.039 (2)	0.033 (2)	0.043 (2)	−0.0001 (16)	−0.0144 (19)	−0.0080 (16)
C19	0.043 (3)	0.046 (3)	0.204 (8)	0.001 (2)	−0.051 (4)	−0.017 (4)
C20	0.054 (3)	0.042 (2)	0.039 (2)	0.0082 (19)	−0.016 (2)	−0.0092 (18)
C21	0.048 (3)	0.036 (2)	0.048 (3)	0.0050 (18)	−0.011 (2)	−0.0088 (18)
C22	0.061 (3)	0.053 (3)	0.062 (3)	0.002 (2)	0.009 (3)	−0.009 (2)
C23	0.068 (4)	0.045 (3)	0.072 (4)	0.000 (2)	−0.008 (3)	−0.015 (2)
C24	0.0218 (18)	0.0287 (17)	0.0164 (16)	−0.0013 (14)	−0.0075 (13)	−0.0049 (13)
C25	0.0261 (19)	0.0309 (18)	0.0157 (16)	−0.0072 (14)	−0.0100 (14)	−0.0006 (13)
C26	0.0256 (19)	0.0351 (18)	0.0184 (16)	−0.0080 (14)	−0.0086 (14)	−0.0035 (14)
C27	0.030 (2)	0.045 (2)	0.0180 (17)	−0.0093 (16)	−0.0027 (15)	−0.0031 (15)
C28	0.040 (2)	0.042 (2)	0.0213 (18)	−0.0153 (18)	−0.0095 (16)	0.0052 (15)
C29	0.044 (2)	0.0287 (18)	0.0283 (19)	−0.0076 (16)	−0.0181 (17)	0.0015 (15)
C30	0.032 (2)	0.0308 (18)	0.0219 (17)	−0.0077 (15)	−0.0135 (15)	−0.0021 (14)
C31	0.028 (2)	0.0372 (19)	0.0188 (17)	−0.0027 (15)	−0.0047 (15)	−0.0062 (14)
C32	0.046 (3)	0.044 (2)	0.029 (2)	−0.0013 (18)	−0.0106 (18)	−0.0112 (17)
C33	0.040 (2)	0.050 (2)	0.037 (2)	0.0043 (19)	−0.0080 (19)	−0.0081 (18)
C34	0.038 (2)	0.0284 (18)	0.0239 (18)	−0.0005 (15)	−0.0117 (16)	0.0000 (14)
C35	0.036 (2)	0.053 (2)	0.042 (2)	−0.0021 (18)	−0.0119 (19)	−0.0112 (19)
C36	0.044 (3)	0.054 (3)	0.061 (3)	−0.002 (2)	−0.015 (2)	−0.027 (2)
C37	0.064 (4)	0.030 (2)	0.152 (6)	0.002 (2)	0.045 (4)	0.000 (3)
C38	0.107 (6)	0.125 (6)	0.067 (4)	0.060 (5)	−0.039 (4)	−0.004 (4)
C39	0.056 (3)	0.049 (3)	0.094 (4)	0.000 (2)	0.021 (3)	−0.027 (3)
C40	0.038 (2)	0.049 (2)	0.044 (2)	−0.0009 (19)	−0.006 (2)	−0.0015 (19)
C41	0.047 (3)	0.051 (3)	0.065 (3)	0.011 (2)	−0.028 (2)	−0.020 (2)
C42	0.035 (2)	0.050 (2)	0.049 (3)	−0.0011 (19)	−0.006 (2)	−0.0002 (19)
C43	0.061 (3)	0.078 (3)	0.049 (3)	−0.006 (3)	−0.020 (3)	−0.010 (2)
C44	0.057 (3)	0.070 (3)	0.068 (3)	−0.003 (3)	−0.028 (3)	−0.003 (3)
C45	0.063 (3)	0.066 (3)	0.062 (3)	−0.010 (3)	−0.006 (3)	−0.028 (3)
C46	0.069 (4)	0.055 (3)	0.090 (4)	0.010 (3)	−0.020 (3)	−0.025 (3)

### Geometric parameters (Å, °)

**Table:**

Ni1—O1	1.969 (2)	C21—C22	1.510 (7)
Ni1—O4^i^	2.011 (2)	C21—C23	1.527 (6)
Ni1—O2	2.018 (2)	C21—H21	1.0000
Ni1—O5^i^	2.030 (2)	C22—H22A	0.9800
Ni1—O4	2.062 (2)	C22—H22B	0.9800
Ni1—O8	2.159 (2)	C22—H22C	0.9800
Ni2—O4^i^	2.040 (2)	C23—H23A	0.9800
Ni2—O2	2.048 (2)	C23—H23B	0.9800
Ni2—O5	2.051 (2)	C23—H23C	0.9800
Ni2—N2	2.051 (3)	C24—H24	0.9500
Ni2—O6	2.111 (2)	C25—C30	1.407 (5)
Ni2—O7	2.137 (2)	C25—C26	1.408 (5)
P1—O9	1.490 (3)	C26—C27	1.398 (5)
P1—C40	1.783 (4)	C26—C31	1.509 (5)
P1—C42	1.785 (4)	C27—C28	1.376 (5)
P1—C41	1.785 (4)	C27—H27	0.9500
P2—O8	1.484 (3)	C28—C29	1.365 (6)
P2—C37	1.789 (6)	C28—H28	0.9500
P2—C38	1.805 (7)	C29—C30	1.388 (5)
P2—C39	1.809 (5)	C29—H29	0.9500
O1—C1	1.306 (4)	C30—C34	1.510 (5)
O2—C9	1.296 (4)	C31—C32	1.522 (5)
O4—Ni1^i^	2.011 (2)	C31—C33	1.526 (5)
O4—Ni2^i^	2.040 (2)	C31—H31	1.0000
O5—Ni1^i^	2.030 (2)	C32—H32A	0.9800
O10—C45	1.392 (6)	C32—H32B	0.9800
O10—C43	1.408 (6)	C32—H32C	0.9800
N1—C11	1.261 (4)	C33—H33A	0.9800
N1—C12	1.434 (5)	C33—H33B	0.9800
N2—C24	1.296 (4)	C33—H33C	0.9800
N2—C25	1.453 (4)	C34—C36	1.518 (5)
C1—C2	1.421 (5)	C34—C35	1.523 (5)
C1—C10	1.459 (5)	C34—H34	1.0000
C2—C3	1.398 (5)	C35—H35A	0.9800
C2—C11	1.457 (5)	C35—H35B	0.9800
C3—C4	1.365 (5)	C35—H35C	0.9800
C3—H3	0.9500	C36—H36A	0.9800
C4—C5	1.415 (5)	C36—H36B	0.9800
C4—H4	0.9500	C36—H36C	0.9800
C5—C6	1.418 (5)	C37—H37A	0.9800
C5—C10	1.435 (5)	C37—H37B	0.9800
C6—C7	1.355 (5)	C37—H37C	0.9800
C6—H6	0.9500	C38—H38A	0.9800
C7—C8	1.418 (5)	C38—H38B	0.9800
C7—H7	0.9500	C38—H38C	0.9800
C8—C9	1.424 (5)	C39—H39A	0.9800
C8—C24	1.445 (4)	C39—H39B	0.9800
C9—C10	1.461 (4)	C39—H39C	0.9800
C11—H11	0.9500	C40—H40A	0.9800
C12—C13	1.403 (5)	C40—H40B	0.9800
C12—C17	1.408 (5)	C40—H40C	0.9800
C13—C14	1.381 (5)	C41—H41A	0.9800
C13—C18	1.517 (5)	C41—H41B	0.9800
C14—C15	1.383 (6)	C41—H41C	0.9800
C14—H14	0.9500	C42—H42A	0.9800
C15—C16	1.369 (6)	C42—H42B	0.9800
C15—H15	0.9500	C42—H42C	0.9800
C16—C17	1.395 (6)	C43—C44	1.506 (7)
C16—H16	0.9500	C43—H43A	0.9900
C17—C21	1.517 (6)	C43—H43B	0.9900
C18—C19	1.511 (6)	C44—H44A	0.9800
C18—C20	1.524 (6)	C44—H44B	0.9800
C18—H18	1.0000	C44—H44C	0.9800
C19—H19A	0.9800	C45—C46	1.447 (8)
C19—H19B	0.9800	C45—H45A	0.9900
C19—H19C	0.9800	C45—H45B	0.9900
C20—H20A	0.9800	C46—H46A	0.9800
C20—H20B	0.9800	C46—H46B	0.9800
C20—H20C	0.9800	C46—H46C	0.9800
			
O1—Ni1—O4^i^	170.15 (10)	C23—C21—H21	107.9
O1—Ni1—O2	89.63 (9)	C21—C22—H22A	109.5
O4^i^—Ni1—O2	83.12 (8)	C21—C22—H22B	109.5
O1—Ni1—O5^i^	92.90 (9)	H22A—C22—H22B	109.5
O4^i^—Ni1—O5^i^	94.05 (9)	C21—C22—H22C	109.5
O2—Ni1—O5^i^	176.36 (9)	H22A—C22—H22C	109.5
O1—Ni1—O4	91.77 (9)	H22B—C22—H22C	109.5
O4^i^—Ni1—O4	82.24 (9)	C21—C23—H23A	109.5
O2—Ni1—O4	94.82 (9)	C21—C23—H23B	109.5
O5^i^—Ni1—O4	82.50 (9)	H23A—C23—H23B	109.5
O1—Ni1—O8	96.00 (10)	C21—C23—H23C	109.5
O4^i^—Ni1—O8	90.31 (9)	H23A—C23—H23C	109.5
O2—Ni1—O8	87.07 (9)	H23B—C23—H23C	109.5
O5^i^—Ni1—O8	95.26 (9)	N2—C24—C8	130.8 (3)
O4—Ni1—O8	172.02 (9)	N2—C24—H24	114.6
O4^i^—Ni2—O2	81.68 (8)	C8—C24—H24	114.6
O4^i^—Ni2—O5	82.54 (9)	C30—C25—C26	121.1 (3)
O2—Ni2—O5	91.68 (9)	C30—C25—N2	119.4 (3)
O4^i^—Ni2—N2	172.09 (10)	C26—C25—N2	119.5 (3)
O2—Ni2—N2	90.47 (9)	C27—C26—C25	117.2 (3)
O5—Ni2—N2	98.75 (10)	C27—C26—C31	118.7 (3)
O4^i^—Ni2—O6	86.77 (9)	C25—C26—C31	124.2 (3)
O2—Ni2—O6	168.45 (9)	C28—C27—C26	122.1 (3)
O5—Ni2—O6	86.64 (9)	C28—C27—H27	119.0
N2—Ni2—O6	101.08 (10)	C26—C27—H27	119.0
O4^i^—Ni2—O7	87.77 (9)	C29—C28—C27	119.7 (3)
O2—Ni2—O7	88.02 (9)	C29—C28—H28	120.2
O5—Ni2—O7	170.24 (9)	C27—C28—H28	120.2
N2—Ni2—O7	91.01 (10)	C28—C29—C30	121.6 (4)
O6—Ni2—O7	91.71 (9)	C28—C29—H29	119.2
O9—P1—C40	112.60 (19)	C30—C29—H29	119.2
O9—P1—C42	111.4 (2)	C29—C30—C25	118.3 (3)
C40—P1—C42	106.6 (2)	C29—C30—C34	119.3 (3)
O9—P1—C41	112.49 (19)	C25—C30—C34	122.3 (3)
C40—P1—C41	107.1 (2)	C26—C31—C32	111.2 (3)
C42—P1—C41	106.2 (2)	C26—C31—C33	111.4 (3)
O8—P2—C37	112.0 (2)	C32—C31—C33	110.6 (3)
O8—P2—C38	111.2 (3)	C26—C31—H31	107.8
C37—P2—C38	112.4 (4)	C32—C31—H31	107.8
O8—P2—C39	111.0 (2)	C33—C31—H31	107.8
C37—P2—C39	106.5 (3)	C31—C32—H32A	109.5
C38—P2—C39	103.3 (3)	C31—C32—H32B	109.5
C1—O1—Ni1	129.6 (2)	H32A—C32—H32B	109.5
C9—O2—Ni1	130.53 (19)	C31—C32—H32C	109.5
C9—O2—Ni2	132.03 (19)	H32A—C32—H32C	109.5
Ni1—O2—Ni2	97.30 (9)	H32B—C32—H32C	109.5
Ni1^i^—O4—Ni2^i^	97.80 (9)	C31—C33—H33A	109.5
Ni1^i^—O4—Ni1	97.76 (9)	C31—C33—H33B	109.5
Ni2^i^—O4—Ni1	97.01 (9)	H33A—C33—H33B	109.5
Ni1^i^—O5—Ni2	97.72 (10)	C31—C33—H33C	109.5
P2—O8—Ni1	133.35 (14)	H33A—C33—H33C	109.5
C45—O10—C43	113.4 (4)	H33B—C33—H33C	109.5
C11—N1—C12	116.6 (3)	C30—C34—C36	112.8 (3)
C24—N2—C25	115.6 (3)	C30—C34—C35	111.6 (3)
C24—N2—Ni2	121.3 (2)	C36—C34—C35	110.2 (3)
C25—N2—Ni2	122.6 (2)	C30—C34—H34	107.3
O1—C1—C2	116.9 (3)	C36—C34—H34	107.3
O1—C1—C10	124.2 (3)	C35—C34—H34	107.3
C2—C1—C10	118.9 (3)	C34—C35—H35A	109.5
C3—C2—C1	120.6 (3)	C34—C35—H35B	109.5
C3—C2—C11	120.9 (3)	H35A—C35—H35B	109.5
C1—C2—C11	118.5 (3)	C34—C35—H35C	109.5
C4—C3—C2	121.6 (3)	H35A—C35—H35C	109.5
C4—C3—H3	119.2	H35B—C35—H35C	109.5
C2—C3—H3	119.2	C34—C36—H36A	109.5
C3—C4—C5	120.3 (3)	C34—C36—H36B	109.5
C3—C4—H4	119.8	H36A—C36—H36B	109.5
C5—C4—H4	119.8	C34—C36—H36C	109.5
C4—C5—C6	118.9 (3)	H36A—C36—H36C	109.5
C4—C5—C10	120.8 (3)	H36B—C36—H36C	109.5
C6—C5—C10	120.3 (3)	P2—C37—H37A	109.5
C7—C6—C5	120.5 (3)	P2—C37—H37B	109.5
C7—C6—H6	119.8	H37A—C37—H37B	109.5
C5—C6—H6	119.8	P2—C37—H37C	109.5
C6—C7—C8	122.4 (3)	H37A—C37—H37C	109.5
C6—C7—H7	118.8	H37B—C37—H37C	109.5
C8—C7—H7	118.8	P2—C38—H38A	109.5
C7—C8—C9	119.4 (3)	P2—C38—H38B	109.5
C7—C8—C24	114.8 (3)	H38A—C38—H38B	109.5
C9—C8—C24	125.8 (3)	P2—C38—H38C	109.5
O2—C9—C8	119.0 (3)	H38A—C38—H38C	109.5
O2—C9—C10	122.0 (3)	H38B—C38—H38C	109.5
C8—C9—C10	119.0 (3)	P2—C39—H39A	109.5
C5—C10—C1	117.6 (3)	P2—C39—H39B	109.5
C5—C10—C9	118.4 (3)	H39A—C39—H39B	109.5
C1—C10—C9	124.0 (3)	P2—C39—H39C	109.5
N1—C11—C2	124.9 (3)	H39A—C39—H39C	109.5
N1—C11—H11	117.5	H39B—C39—H39C	109.5
C2—C11—H11	117.5	P1—C40—H40A	109.5
C13—C12—C17	121.2 (3)	P1—C40—H40B	109.5
C13—C12—N1	119.4 (3)	H40A—C40—H40B	109.5
C17—C12—N1	119.4 (3)	P1—C40—H40C	109.5
C14—C13—C12	118.2 (4)	H40A—C40—H40C	109.5
C14—C13—C18	120.7 (4)	H40B—C40—H40C	109.5
C12—C13—C18	121.1 (3)	P1—C41—H41A	109.5
C13—C14—C15	121.6 (4)	P1—C41—H41B	109.5
C13—C14—H14	119.2	H41A—C41—H41B	109.5
C15—C14—H14	119.2	P1—C41—H41C	109.5
C16—C15—C14	119.8 (4)	H41A—C41—H41C	109.5
C16—C15—H15	120.1	H41B—C41—H41C	109.5
C14—C15—H15	120.1	P1—C42—H42A	109.5
C15—C16—C17	121.4 (4)	P1—C42—H42B	109.5
C15—C16—H16	119.3	H42A—C42—H42B	109.5
C17—C16—H16	119.3	P1—C42—H42C	109.5
C16—C17—C12	117.9 (4)	H42A—C42—H42C	109.5
C16—C17—C21	121.4 (4)	H42B—C42—H42C	109.5
C12—C17—C21	120.7 (4)	O10—C43—C44	108.9 (4)
C19—C18—C13	109.7 (3)	O10—C43—H43A	109.9
C19—C18—C20	110.5 (4)	C44—C43—H43A	109.9
C13—C18—C20	113.8 (3)	O10—C43—H43B	109.9
C19—C18—H18	107.5	C44—C43—H43B	109.9
C13—C18—H18	107.5	H43A—C43—H43B	108.3
C20—C18—H18	107.5	C43—C44—H44A	109.5
C18—C19—H19A	109.5	C43—C44—H44B	109.5
C18—C19—H19B	109.5	H44A—C44—H44B	109.5
H19A—C19—H19B	109.5	C43—C44—H44C	109.5
C18—C19—H19C	109.5	H44A—C44—H44C	109.5
H19A—C19—H19C	109.5	H44B—C44—H44C	109.5
H19B—C19—H19C	109.5	O10—C45—C46	110.1 (4)
C18—C20—H20A	109.5	O10—C45—H45A	109.6
C18—C20—H20B	109.5	C46—C45—H45A	109.6
H20A—C20—H20B	109.5	O10—C45—H45B	109.6
C18—C20—H20C	109.5	C46—C45—H45B	109.6
H20A—C20—H20C	109.5	H45A—C45—H45B	108.1
H20B—C20—H20C	109.5	C45—C46—H46A	109.5
C22—C21—C17	110.9 (4)	C45—C46—H46B	109.5
C22—C21—C23	109.7 (4)	H46A—C46—H46B	109.5
C17—C21—C23	112.4 (4)	C45—C46—H46C	109.5
C22—C21—H21	107.9	H46A—C46—H46C	109.5
C17—C21—H21	107.9	H46B—C46—H46C	109.5
			
O2—Ni1—O1—C1	2.6 (3)	C24—C8—C9—C10	179.9 (3)
O5^i^—Ni1—O1—C1	−174.8 (3)	C4—C5—C10—C1	−2.3 (5)
O4—Ni1—O1—C1	−92.2 (3)	C6—C5—C10—C1	176.6 (3)
O8—Ni1—O1—C1	89.6 (3)	C4—C5—C10—C9	177.0 (3)
O1—Ni1—O2—C9	0.4 (3)	C6—C5—C10—C9	−4.0 (5)
O4^i^—Ni1—O2—C9	173.7 (3)	O1—C1—C10—C5	−179.1 (3)
O4—Ni1—O2—C9	92.1 (3)	C2—C1—C10—C5	0.7 (4)
O8—Ni1—O2—C9	−95.6 (3)	O1—C1—C10—C9	1.6 (5)
O1—Ni1—O2—Ni2	−175.67 (10)	C2—C1—C10—C9	−178.6 (3)
O4^i^—Ni1—O2—Ni2	−2.36 (9)	O2—C9—C10—C5	−177.9 (3)
O4—Ni1—O2—Ni2	−83.93 (10)	C8—C9—C10—C5	1.8 (5)
O8—Ni1—O2—Ni2	88.30 (10)	O2—C9—C10—C1	1.4 (5)
O4^i^—Ni2—O2—C9	−173.6 (3)	C8—C9—C10—C1	−178.9 (3)
O5—Ni2—O2—C9	−91.4 (3)	C12—N1—C11—C2	−179.9 (3)
N2—Ni2—O2—C9	7.3 (3)	C3—C2—C11—N1	8.0 (5)
O6—Ni2—O2—C9	−172.8 (4)	C1—C2—C11—N1	−171.1 (3)
O7—Ni2—O2—C9	98.3 (3)	C11—N1—C12—C13	−91.9 (4)
O4^i^—Ni2—O2—Ni1	2.34 (9)	C11—N1—C12—C17	89.6 (4)
O5—Ni2—O2—Ni1	84.54 (10)	C17—C12—C13—C14	−0.4 (5)
N2—Ni2—O2—Ni1	−176.69 (11)	N1—C12—C13—C14	−178.8 (3)
O6—Ni2—O2—Ni1	3.1 (5)	C17—C12—C13—C18	178.4 (3)
O7—Ni2—O2—Ni1	−85.70 (10)	N1—C12—C13—C18	−0.1 (5)
O1—Ni1—O4—Ni1^i^	172.15 (9)	C12—C13—C14—C15	0.3 (6)
O4^i^—Ni1—O4—Ni1^i^	0.0	C18—C13—C14—C15	−178.5 (4)
O2—Ni1—O4—Ni1^i^	82.37 (9)	C13—C14—C15—C16	0.0 (7)
O5^i^—Ni1—O4—Ni1^i^	−95.15 (10)	C14—C15—C16—C17	−0.1 (7)
O1—Ni1—O4—Ni2^i^	−88.95 (9)	C15—C16—C17—C12	0.0 (6)
O4^i^—Ni1—O4—Ni2^i^	98.90 (10)	C15—C16—C17—C21	−177.0 (4)
O2—Ni1—O4—Ni2^i^	−178.73 (8)	C13—C12—C17—C16	0.3 (6)
O5^i^—Ni1—O4—Ni2^i^	3.75 (8)	N1—C12—C17—C16	178.7 (3)
O4^i^—Ni2—O5—Ni1^i^	−3.78 (8)	C13—C12—C17—C21	177.3 (3)
O2—Ni2—O5—Ni1^i^	−85.16 (9)	N1—C12—C17—C21	−4.2 (5)
N2—Ni2—O5—Ni1^i^	−175.89 (9)	C14—C13—C18—C19	89.3 (5)
O6—Ni2—O5—Ni1^i^	83.40 (9)	C12—C13—C18—C19	−89.5 (5)
C37—P2—O8—Ni1	−55.4 (3)	C14—C13—C18—C20	−35.1 (5)
C38—P2—O8—Ni1	71.3 (3)	C12—C13—C18—C20	146.2 (4)
C39—P2—O8—Ni1	−174.3 (3)	C16—C17—C21—C22	71.9 (5)
O1—Ni1—O8—P2	40.0 (2)	C12—C17—C21—C22	−105.0 (5)
O4^i^—Ni1—O8—P2	−147.6 (2)	C16—C17—C21—C23	−51.3 (6)
O2—Ni1—O8—P2	129.3 (2)	C12—C17—C21—C23	131.7 (4)
O5^i^—Ni1—O8—P2	−53.5 (2)	C25—N2—C24—C8	−176.5 (3)
O2—Ni2—N2—C24	−1.4 (3)	Ni2—N2—C24—C8	−4.2 (5)
O5—Ni2—N2—C24	90.3 (3)	C7—C8—C24—N2	−175.0 (3)
O6—Ni2—N2—C24	178.6 (2)	C9—C8—C24—N2	6.6 (6)
O7—Ni2—N2—C24	−89.5 (3)	C24—N2—C25—C30	−108.6 (3)
O2—Ni2—N2—C25	170.3 (2)	Ni2—N2—C25—C30	79.3 (3)
O5—Ni2—N2—C25	−97.9 (2)	C24—N2—C25—C26	71.7 (4)
O6—Ni2—N2—C25	−9.7 (3)	Ni2—N2—C25—C26	−100.4 (3)
O7—Ni2—N2—C25	82.3 (2)	C30—C25—C26—C27	0.9 (5)
Ni1—O1—C1—C2	176.4 (2)	N2—C25—C26—C27	−179.4 (3)
Ni1—O1—C1—C10	−3.8 (5)	C30—C25—C26—C31	−179.8 (3)
O1—C1—C2—C3	−178.3 (3)	N2—C25—C26—C31	−0.1 (5)
C10—C1—C2—C3	1.9 (5)	C25—C26—C27—C28	−0.9 (5)
O1—C1—C2—C11	0.8 (4)	C31—C26—C27—C28	179.7 (3)
C10—C1—C2—C11	−179.0 (3)	C26—C27—C28—C29	0.6 (5)
C1—C2—C3—C4	−3.1 (5)	C27—C28—C29—C30	−0.2 (6)
C11—C2—C3—C4	177.9 (3)	C28—C29—C30—C25	0.2 (5)
C2—C3—C4—C5	1.4 (5)	C28—C29—C30—C34	177.4 (3)
C3—C4—C5—C6	−177.7 (3)	C26—C25—C30—C29	−0.6 (5)
C3—C4—C5—C10	1.3 (5)	N2—C25—C30—C29	179.7 (3)
C4—C5—C6—C7	−178.1 (3)	C26—C25—C30—C34	−177.7 (3)
C10—C5—C6—C7	2.9 (5)	N2—C25—C30—C34	2.6 (5)
C5—C6—C7—C8	0.6 (5)	C27—C26—C31—C32	67.1 (4)
C6—C7—C8—C9	−2.8 (5)	C25—C26—C31—C32	−112.2 (4)
C6—C7—C8—C24	178.7 (3)	C27—C26—C31—C33	−56.9 (4)
Ni1—O2—C9—C8	178.2 (2)	C25—C26—C31—C33	123.8 (4)
Ni2—O2—C9—C8	−7.0 (4)	C29—C30—C34—C36	57.8 (4)
Ni1—O2—C9—C10	−2.1 (4)	C25—C30—C34—C36	−125.0 (4)
Ni2—O2—C9—C10	172.6 (2)	C29—C30—C34—C35	−66.9 (4)
C7—C8—C9—O2	−178.8 (3)	C25—C30—C34—C35	110.2 (4)
C24—C8—C9—O2	−0.4 (5)	C45—O10—C43—C44	−176.5 (4)
C7—C8—C9—C10	1.5 (5)	C43—O10—C45—C46	−175.0 (4)

Symmetry codes: (i) −*x*, −*y*+1, −*z*+1.
